# Supplementary material for: Variational Polaron Theory for Ground States of Strongly Coupled Light–Matter and Electron–Phonon Systems
Source: J Phys Chem Lett. 2026 Sep 21;17(39):11162–70. doi: 10.1021/acs.jpclett.6c02011 (PMC13637079; doi:10.1021/acs.jpclett.6c02011)
Supplement: Supplementary file 1 [file jz6c02011_si_001.pdf]

Supporting Information

Variational Polaron Theory for Ground States of Strongly Coupled Light–Matter and Electron–Phonon Systems

Nguyen Thanh Phuc
Department of Chemical Science and Engineering,
Graduate School of Engineering, Kyoto University, Kyoto 615-8510, Japan

S1 Transformation of Off-Diagonal Matter Operators

In this section, we derive how an off-diagonal matter operator is transformed under the state-dependent polaron transformation. We assume that the matter projectors $\{\hat{P}_\mu\}$ are orthogonal and complete,

$$\hat{P}_\mu \hat{P}_\nu = \delta_{\mu\nu}\hat{P}_\mu, \qquad \sum_\mu \hat{P}_\mu = \hat{I}_{\mathrm{M}}. \tag{S1}$$

We define

$$\hat{X}_\alpha = \hat{b}_\alpha^\dagger - \hat{b}_\alpha, \qquad \hat{A}_\mu = \sum_\alpha \lambda_{\alpha,\mu}\hat{X}_\alpha, \tag{S2}$$

where $\lambda_{\alpha,\mu}$ is the displacement of bosonic mode α associated with matter sector μ. The state-dependent polaron transformation is then

$$\hat{U} = \exp\left[\sum_{\alpha,\mu} \lambda_{\alpha,\mu}\hat{P}_\mu\left(\hat{b}_\alpha^\dagger - \hat{b}_\alpha\right)\right] = \exp\left[\sum_\mu \hat{P}_\mu \hat{A}_\mu\right]. \tag{S3}$$

Because the projectors are mutually orthogonal, the exponential is block diagonal in the matter sectors:

$$\hat{U} = \sum_\mu \hat{P}_\mu e^{\hat{A}_\mu}, \qquad \hat{U}^\dagger = \sum_\mu \hat{P}_\mu e^{-\hat{A}_\mu}. \tag{S4}$$

We now consider a matter operator that connects sector ν to sector μ,

$$\hat{O}_{\mu\nu} = \hat{P}_\mu \hat{O} \hat{P}_\nu. \tag{S5}$$

Using Eq. (S4), we obtain

$$\hat{U}^\dagger \hat{O}_{\mu\nu}\hat{U} = \left(\sum_\rho \hat{P}_\rho e^{-\hat{A}_\rho}\right)\hat{P}_\mu \hat{O}\hat{P}_\nu \left(\sum_\sigma \hat{P}_\sigma e^{\hat{A}_\sigma}\right). \tag{S6}$$

The orthogonality relations $\hat{P}_\rho \hat{P}_\mu = \delta_{\rho\mu}\hat{P}_\mu$ and $\hat{P}_\nu \hat{P}_\sigma = \delta_{\nu\sigma}\hat{P}_\nu$ select a single term from the sums, giving

$$\hat{U}^\dagger \hat{O}_{\mu\nu}\hat{U} = \hat{P}_\mu e^{-\hat{A}_\mu}\hat{O}\hat{P}_\nu e^{\hat{A}_\nu}. \tag{S7}$$

Since $\hat{O}$, $\hat{P}_\mu$, and $\hat{P}_\nu$ act only on the matter Hilbert space, whereas $e^{\pm\hat{A}_\mu}$ act only on the bosonic Hilbert space, these operators commute with one another. Therefore,

$$\begin{aligned}\hat{U}^\dagger\hat{O}_{\mu\nu}\hat{U} &= \hat{P}_\mu\hat{O}\hat{P}_\nu e^{-\hat{A}_\mu}e^{\hat{A}_\nu} \\ &= \hat{O}_{\mu\nu}e^{\hat{A}_\nu-\hat{A}_\mu}.\end{aligned} \tag{S8}$$

Substituting the definition of $\hat{A}_\mu$, we find

$$\hat{A}_\nu - \hat{A}_\mu = \sum_\alpha (\lambda_{\alpha,\nu} - \lambda_{\alpha,\mu})\left(\hat{b}_\alpha^\dagger - \hat{b}_\alpha\right). \tag{S9}$$

For different bosonic modes,

$$\left[\hat{b}_\alpha^\dagger - \hat{b}_\alpha, \hat{b}_\beta^\dagger - \hat{b}_\beta\right] = 0 \qquad (\alpha \neq \beta), \tag{S10}$$

and hence the exponential factorizes into a product over modes:

$$e^{\hat{A}_\nu-\hat{A}_\mu} = \prod_\alpha \exp\left[(\lambda_{\alpha,\nu} - \lambda_{\alpha,\mu})\left(\hat{b}_\alpha^\dagger - \hat{b}_\alpha\right)\right]. \tag{S11}$$

Thus, the transformed off-diagonal matter operator is

$$\hat{U}^\dagger\hat{O}_{\mu\nu}\hat{U} = \hat{O}_{\mu\nu}\prod_\alpha \exp\left[(\lambda_{\alpha,\nu} - \lambda_{\alpha,\mu})\left(\hat{b}_\alpha^\dagger - \hat{b}_\alpha\right)\right]. \tag{S12}$$

Equation (S12) shows that an off-diagonal matter transition is accompanied by a product of displacement operators whose amplitudes are given by the differences between the bosonic displacements of the two matter sectors. Therefore, off-diagonal matter couplings in the transformed frame are controlled by overlaps between displaced bosonic states. When the displacement differences grow in the strong-coupling limit, these overlaps are exponentially suppressed, leading to asymptotic decoupling of the transformed Hamiltonian.

S2 Variational polaron theory for the Dicke model

This section summarizes the equations of the variational polaron theory for the Dicke model.

S2.1 Hamiltonian and spin basis

The Dicke Hamiltonian without the rotating-wave approximation is

$$\hat{H}_\mathrm{D} = \omega\hat{a}^\dagger\hat{a} + \omega_0\hat{S}_z + g(\hat{a}^\dagger + \hat{a})\hat{S}_x, \tag{S13}$$

where

$$\hat{S}_\alpha = \sum_{j=1}^N \hat{s}_\alpha^{(j)} = \frac{1}{2}\sum_{j=1}^N \hat{\sigma}_\alpha^{(j)}. \tag{S14}$$

Because $[\hat{H}_\mathrm{D}, \hat{\mathbf{S}}^2] = 0$ with $\hat{\mathbf{S}}^2 = \sum_{\alpha=x,y,z}\hat{S}_\alpha^2$ and all emitters are equivalent, the ground state lies in the maximal-spin sector $S = N/2$. It is convenient to rotate the spin axes and write the Dicke Hamiltonian as

$$\hat{H}_\mathrm{D} = \omega\hat{a}^\dagger\hat{a} + \omega_0\hat{S}_x + g(\hat{a}^\dagger + \hat{a})\hat{S}_z. \tag{S15}$$

Depending on the convention used for the spin rotation, the light–matter coupling changes sign. In the following, we absorb this possible sign change into the definition of g and use g throughout the derivation. This sign convention does not affect the spectrum or the variational results. We work in the fully symmetric spin manifold $S = N/2$ and use the eigenstates of $\hat{S}_z$,

$$|l\rangle \equiv |S = N/2, S_z = l\rangle , \qquad l = -N/2, \ldots, N/2, \tag{S16}$$

with the corresponding projectors

$$\hat{P}_l = |l\rangle \langle l| . \tag{S17}$$

The spin ladder matrix elements are

$$\hat{S}_+ |l\rangle = s_l |l+1\rangle , \qquad \hat{S}_- |l+1\rangle = s_l |l\rangle , \tag{S18}$$

where

$$s_l = \sqrt{\frac{N}{2}\left(\frac{N}{2}+1\right) - l(l+1)}. \tag{S19}$$

S2.2 State-dependent displacement and transformed Hamiltonian

We use the variational polaron transformation

$$\hat{U}_{\mathrm{D}}(\boldsymbol{\lambda}) = \exp\left[(\hat{a}^\dagger - \hat{a})\sum_l \lambda_l \hat{P}_l\right]. \tag{S20}$$

With this sign convention,

$$\hat{U}_{\mathrm{D}}^\dagger \hat{a} \hat{U}_{\mathrm{D}} = \hat{a} + \sum_l \lambda_l \hat{P}_l, \qquad \hat{U}_{\mathrm{D}}^\dagger \hat{S}_z \hat{U}_{\mathrm{D}} = \hat{S}_z. \tag{S21}$$

The raising and lowering operators transform as

$$\hat{U}_{\mathrm{D}}^\dagger \hat{S}_+ \hat{U}_{\mathrm{D}} = \sum_{l=-N/2}^{N/2-1} s_l \exp\left[-(\hat{a}^\dagger - \hat{a})(\lambda_{l+1} - \lambda_l)\right] |l+1\rangle \langle l| , \tag{S22}$$

$$\hat{U}_{\mathrm{D}}^\dagger \hat{S}_- \hat{U}_{\mathrm{D}} = \sum_{l=-N/2}^{N/2-1} s_l \exp\left[-(\hat{a}^\dagger - \hat{a})(\lambda_l - \lambda_{l+1})\right] |l\rangle \langle l+1| . \tag{S23}$$

The transformed Hamiltonian $\hat{H}_{\mathrm{D}}(\boldsymbol{\lambda}) = \hat{U}_{\mathrm{D}}^\dagger \hat{H}_{\mathrm{D}} \hat{U}_{\mathrm{D}}$ is therefore

$$\begin{aligned}\hat{H}_{\mathrm{D}}(\boldsymbol{\lambda}) =& \omega \hat{a}^\dagger \hat{a} + (\hat{a}^\dagger + \hat{a})\sum_l (\omega\lambda_l + gl)\hat{P}_l + \sum_l (\omega\lambda_l^2 + 2gl\lambda_l)\hat{P}_l \\ &+ \frac{\omega_0}{2}\sum_{l=-N/2}^{N/2-1} s_l \Big[e^{-(\hat{a}^\dagger - \hat{a})(\lambda_{l+1}-\lambda_l)} |l+1\rangle \langle l| + e^{-(\hat{a}^\dagger - \hat{a})(\lambda_l - \lambda_{l+1})} |l\rangle \langle l+1|\Big].\end{aligned} \tag{S24}$$

The linear light–matter force is canceled in the strong-coupling limit by $\lambda_l \simeq -gl/\omega$ with the present sign convention. Off-diagonal spin transitions are then suppressed by the displacement overlaps in Eqs. (S30) and (S31) whenever $|\lambda_{l+1} - \lambda_l|$ becomes large.

S2.3 Variational energy in the transformed frame

The zeroth-order product state is

$$|\Phi_0\rangle = |\Phi_0^{\mathrm{S}}\rangle \otimes |\Phi_0^{\mathrm{B}}\rangle, \qquad |\Phi_0^{\mathrm{S}}\rangle = \sum_l A_l |l\rangle, \qquad |\Phi_0^{\mathrm{B}}\rangle = \sum_{n=0}^{N_{\mathrm{cut}}} c_n |n\rangle, \tag{S25}$$

where the coefficients are real for the ground state. In this subsection, the coefficients A_l and c_n are not assumed to be normalized during the variational differentiation. The unnormalized energy expectation value is

$$\begin{aligned}\mathcal{E}_{\mathrm{D}} &\equiv \langle\Phi_0| \hat{H}_{\mathrm{D}}(\boldsymbol{\lambda}) |\Phi_0\rangle \\ &= \omega\left(\sum_l A_l^2\right)\left(\sum_n n c_n^2\right) + 2\left(\sum_n \sqrt{n+1}\, c_n c_{n+1}\right)\left(\sum_l (\omega\lambda_l + gl) A_l^2\right) \\ &\quad + \left(\sum_n c_n^2\right)\left[\sum_l \left(\omega\lambda_l^2 + 2gl\lambda_l\right) A_l^2\right] + \omega_0 \sum_{l=-N/2}^{N/2-1} s_l A_l A_{l+1} \sum_{n,m=0}^{N_{\mathrm{cut}}} c_n c_m D_{nm}^{\lambda_l-\lambda_{l+1}}.\end{aligned} \tag{S26}$$

The norm is

$$\mathcal{N}_{\mathrm{D}} = \langle\Phi_0|\Phi_0\rangle = \left(\sum_l A_l^2\right)\left(\sum_n c_n^2\right), \tag{S27}$$

and the normalized variational energy is

$$E_{\mathrm{var}}^{\mathrm{D}} = \frac{\mathcal{E}_{\mathrm{D}}}{\mathcal{N}_{\mathrm{D}}}. \tag{S28}$$

Here, the matrix element of a displacement operator between Fock states is denoted by

$$D_{nm}^{\lambda} = \langle n| e^{\lambda(\hat{b}^\dagger - \hat{b})} |m\rangle. \tag{S29}$$

For real λ,

$$D_{nm}^{\lambda} = \sqrt{\frac{m!}{n!}}\, e^{-\lambda^2/2}\lambda^{n-m} L_m^{n-m}(\lambda^2), \qquad n \geq m, \tag{S30}$$

$$D_{nm}^{\lambda} = \sqrt{\frac{n!}{m!}}\, e^{-\lambda^2/2}(-\lambda)^{m-n} L_n^{m-n}(\lambda^2), \qquad n \leq m, \tag{S31}$$

where $L_n^k(x)$ is an associated Laguerre polynomial,

$$L_n^k(x) = \sum_{r=0}^{n} (-1)^r \binom{n+k}{r+k} \frac{x^r}{r!}. \tag{S32}$$

Note that $D_{nm}^{\lambda} = D_{mn}^{-\lambda}$. For fixed finite n, m, $D_{nm}^{\lambda} \to 0$ as $|\lambda| \to \infty$, because the Laguerre polynomial grows algebraically whereas $e^{-\lambda^2/2}$ decays exponentially.

The parameters $\{A_l, c_n, \lambda_l\}$ are optimized by gradient descent. Since the variational energy is a ratio, its derivative with respect to any real variational parameter $x \in \{\lambda_l, A_l, c_n\}$ is

$$\frac{\partial E_{\mathrm{var}}^{\mathrm{D}}}{\partial x} = \frac{\mathcal{N}_{\mathrm{D}}\left(\partial\mathcal{E}_{\mathrm{D}}/\partial x\right) - \mathcal{E}_{\mathrm{D}}\left(\partial\mathcal{N}_{\mathrm{D}}/\partial x\right)}{\mathcal{N}_{\mathrm{D}}^2}. \tag{S33}$$

With the convention that terms with indices outside the allowed ranges are omitted, the derivatives of the unnormalized numerator are as follows. The derivative with respect to the displacement parameter is

$$\frac{\partial \mathcal{E}_{\rm D}}{\partial \lambda_l} = 2(\omega\lambda_l + gl)A_l^2\left(\sum_n c_n^2\right) + 2\omega A_l^2\left(\sum_n c_n c_{n+1}\sqrt{n+1}\right) + \omega_0 \sum_{n,m} c_n c_m \left[A_l A_{l+1}\, \frac{\partial D^{\lambda}_{mn}}{\partial \lambda}\bigg|_{\lambda=\lambda_l-\lambda_{l+1}} s_l - A_{l-1}A_l\, \frac{\partial D^{\lambda}_{mn}}{\partial \lambda}\bigg|_{\lambda=\lambda_{l-1}-\lambda_l} s_{l-1}\right]. \tag{S34}$$

The derivative with respect to the spin coefficient is

$$\frac{\partial \mathcal{E}_{\rm D}}{\partial A_l} = 2\omega A_l\left(\sum_n n c_n^2\right) + 2\left(\omega\lambda_l^2 + 2gl\lambda_l\right)A_l\left(\sum_n c_n^2\right) + 4(\omega\lambda_l + gl)A_l\left(\sum_n c_n c_{n+1}\sqrt{n+1}\right) + \omega_0\sum_{n,m} c_n c_m\left[A_{l+1}D_{mn}^{\lambda_l-\lambda_{l+1}} s_l + A_{l-1}D_{mn}^{\lambda_{l-1}-\lambda_l} s_{l-1}\right]. \tag{S35}$$

The derivative with respect to the bosonic coefficient is

$$\frac{\partial \mathcal{E}_{\rm D}}{\partial c_n} = 2\omega n c_n\left(\sum_l A_l^2\right) + 2\omega c_n\left(\sum_l \lambda_l^2 A_l^2\right) + 4g c_n\left(\sum_l l\lambda_l A_l^2\right) + 2\left(c_{n+1}\sqrt{n+1} + c_{n-1}\sqrt{n}\right)\left[\sum_l(\omega\lambda_l + gl)A_l^2\right] + \omega_0 \sum_{l=-N/2}^{N/2-1}\sum_m A_l A_{l+1} c_m\left(D_{mn}^{\lambda_l-\lambda_{l+1}} + D_{nm}^{\lambda_l-\lambda_{l+1}}\right)s_l. \tag{S36}$$

Here $c_{-1} = c_{N_{\rm cut}+1} = 0$. The derivatives of the norm are

$$\frac{\partial \mathcal{N}_{\rm D}}{\partial \lambda_l} = 0, \tag{S37}$$

$$\frac{\partial \mathcal{N}_{\rm D}}{\partial A_l} = 2A_l\left(\sum_n c_n^2\right), \tag{S38}$$

$$\frac{\partial \mathcal{N}_{\rm D}}{\partial c_n} = 2c_n\left(\sum_l A_l^2\right). \tag{S39}$$

After each gradient-descent step, the coefficients are renormalized so that $\sum_l A_l^2 = 1$ and $\sum_n c_n^2 = 1$. Even with this post-step renormalization, the gradients used in the update are those of the normalized energy ratio in Eq. (S33).

S2.4 Second-order perturbation theory for the Dicke model

Define the spin and bosonic mean-field Hamiltonians by

$$\hat{H}_{\rm S} = \left\langle \Phi_0^{\rm B}\right| \hat{H}_{\rm D}(\boldsymbol{\lambda}) \left|\Phi_0^{\rm B}\right\rangle, \qquad \hat{H}_{\rm B} = \left\langle \Phi_0^{\rm S}\right| \hat{H}_{\rm D}(\boldsymbol{\lambda}) \left|\Phi_0^{\rm S}\right\rangle, \tag{S40}$$

and

$$\hat{H}_0^{\rm D} = \hat{H}_{\rm S} + \hat{H}_{\rm B} - E_0, \qquad E_0 = \langle \Phi_0 | \hat{H}_{\rm D}(\boldsymbol{\lambda}) | \Phi_0\rangle. \tag{S41}$$

Let

$$\hat{H}_{\rm S}\left|\Phi_p^{\rm S}\right\rangle = \epsilon_p^{\rm S}\left|\Phi_p^{\rm S}\right\rangle, \qquad \hat{H}_{\rm B}\left|\Phi_q^{\rm B}\right\rangle = \epsilon_q^{\rm B}\left|\Phi_q^{\rm B}\right\rangle. \tag{S42}$$

Then

$$|\Phi_{pq}\rangle = \left|\Phi_p^{\rm S}\right\rangle \otimes \left|\Phi_q^{\rm B}\right\rangle, \qquad E_{pq} = \epsilon_p^{\rm S} + \epsilon_q^{\rm B} - E_0. \tag{S43}$$

The residual Hamiltonian is

$$\delta\hat{H}^{\rm D} = \hat{H}_{\rm D}(\boldsymbol{\lambda}) - \hat{H}_0^{\rm D}. \tag{S44}$$

The second-order energy correction and first-order corrected state are

$$E_0^{(2)} = \sum_{(p,q)\neq(0,0)} \frac{|\langle\Phi_{pq}|\,\delta\hat{H}^{\rm D}\,|\Phi_{00}\rangle|^2}{E_{00} - E_{pq}}, \tag{S45}$$

$$\left|\psi_0^{(1)}\right\rangle = |\Phi_{00}\rangle + \sum_{(p,q)\neq(0,0)} \frac{\langle\Phi_{pq}|\,\delta\hat{H}^{\rm D}\,|\Phi_{00}\rangle}{E_{00} - E_{pq}}\,|\Phi_{pq}\rangle. \tag{S46}$$

In the calculations reported in the main text, this perturbative correction reduces the Dicke-model energy error and improves the ground-state fidelity over the full range of collective coupling.

S3 Holstein model in a transformed Fock basis

This section gives the working equations for the one-electron Holstein benchmark in the Fock-state representation of the residual phonons.

S3.1 Hamiltonian and polaron transformation

We consider a one-dimensional spinless single-electron Holstein Hamiltonian,

$$\hat{H}_{\rm H} = -J\sum_{\langle i,j\rangle}\left(\hat{c}_i^\dagger\hat{c}_j + \hat{c}_j^\dagger\hat{c}_i\right) + \omega\sum_i \hat{b}_i^\dagger\hat{b}_i + g\sum_i \hat{c}_i^\dagger\hat{c}_i(\hat{b}_i^\dagger + \hat{b}_i). \tag{S47}$$

The state-dependent polaron transformation is

$$\hat{U}_{\rm H}(\boldsymbol{\lambda}) = \exp\left[\sum_i \lambda_i\hat{c}_i^\dagger\hat{c}_i(\hat{b}_i^\dagger - \hat{b}_i)\right], \tag{S48}$$

with real displacement parameters λ_i. The transformed operators are

$$\hat{U}_{\rm H}^\dagger\hat{b}_i\hat{U}_{\rm H} = \hat{b}_i + \lambda_i\hat{c}_i^\dagger\hat{c}_i, \tag{S49}$$

$$\hat{U}_{\rm H}^\dagger\hat{c}_i\hat{U}_{\rm H} = \hat{c}_i\exp\left[\lambda_i(\hat{b}_i^\dagger - \hat{b}_i)\right]. \tag{S50}$$

The transformed Hamiltonian $\hat{H}_{\rm H}(\boldsymbol{\lambda}) = \hat{U}_{\rm H}^\dagger\hat{H}_{\rm H}\hat{U}_{\rm H}$ is

$$\begin{aligned}\hat{H}_{\rm H}(\boldsymbol{\lambda}) = &- J\sum_{\langle i,j\rangle}\left[\hat{c}_i^\dagger\hat{c}_j e^{-\lambda_i(\hat{b}_i^\dagger-\hat{b}_i)}e^{\lambda_j(\hat{b}_j^\dagger-\hat{b}_j)} + \hat{c}_j^\dagger\hat{c}_i e^{-\lambda_j(\hat{b}_j^\dagger-\hat{b}_j)}e^{\lambda_i(\hat{b}_i^\dagger-\hat{b}_i)}\right]\\ &+ \omega\sum_i \hat{b}_i^\dagger\hat{b}_i + \sum_i(\omega\lambda_i + g)(\hat{b}_i^\dagger + \hat{b}_i)\hat{c}_i^\dagger\hat{c}_i + \sum_i \lambda_i(\omega\lambda_i + 2g)\hat{c}_i^\dagger\hat{c}_i\hat{c}_i^\dagger\hat{c}_i.\end{aligned} \tag{S51}$$

For a single electron, $\hat{c}_i^\dagger\hat{c}_i\hat{c}_i^\dagger\hat{c}_i = \hat{c}_i^\dagger\hat{c}_i$ within the one-particle sector.

S3.2 Variational energy in the transformed frame

The electronic and phononic states in the transformed frame are expanded as

$$\left|\Phi_0^{\mathrm{E}}\right\rangle = \sum_{i=1}^{N_{\mathrm{site}}} A_i \left|i\right\rangle , \tag{S52}$$

$$\left|\Phi_0^{\mathrm{P}}\right\rangle = \sum_{n_1,\ldots,n_{N_{\mathrm{site}}}=0}^{N_{\mathrm{cut}}} c_{n_1,\ldots,n_{N_{\mathrm{site}}}} \left|n_1,\ldots,n_{N_{\mathrm{site}}}\right\rangle , \tag{S53}$$

with real coefficients for the ground state. The zeroth-order product state is

$$\left|\Phi_0\right\rangle = \left|\Phi_0^{\mathrm{E}}\right\rangle \otimes \left|\Phi_0^{\mathrm{P}}\right\rangle . \tag{S54}$$

The unnormalized energy expectation value in the transformed frame is

$$\mathcal{E}_{\mathrm{H}} \equiv \left\langle\Phi_0\right| \hat{H}_{\mathrm{H}}(\boldsymbol{\lambda}) \left|\Phi_0\right\rangle . \tag{S55}$$

For compact notation, we write $\mathbf{n} = (n_1, \ldots, n_{N_{\mathrm{site}}})$, and denote by $\mathbf{n}_{ij}(m_i, m_j)$ the phonon configuration obtained from $\mathbf{n}$ by replacing n_i and n_j with m_i and m_j, respectively. Then

$$\begin{aligned}\mathcal{E}_{\mathrm{H}} = &- J \sum_{\langle i,j\rangle} A_i A_j \sum_{\mathbf{n}} \sum_{m_i,m_j} c_{\mathbf{n}} c_{\mathbf{n}_{ij}(m_i,m_j)} \left(D^{\lambda_i}_{m_i n_i} D^{-\lambda_j}_{m_j n_j} + D^{\lambda_j}_{m_j n_j} D^{-\lambda_i}_{m_i n_i} \right) \\ &+ \omega \left(\sum_i A_i^2 \right) \sum_{\mathbf{n}} \left(\sum_i n_i \right) c_{\mathbf{n}}^2 + 2 \sum_i (\omega\lambda_i + g) A_i^2 \sum_{\mathbf{n}} \sqrt{n_i + 1}\, c_{\mathbf{n}} c_{\mathbf{n}+\mathbf{e}_i} \\ &+ \left(\sum_{\mathbf{n}} c_{\mathbf{n}}^2 \right) \sum_i \lambda_i (\omega\lambda_i + 2g) A_i^2. \end{aligned} \tag{S56}$$

Here, $\mathbf{e}_i$ is the unit vector that increases n_i by one, and coefficients with indices outside the retained phonon Hilbert space are taken to be zero.

The derivative with respect to the displacement parameter λ_i is

$$\begin{aligned}\frac{\partial \mathcal{E}_{\mathrm{H}}}{\partial \lambda_i} = &- J \sum_{j\in\mathrm{nn}(i)} A_i A_j \sum_{\mathbf{n}} \sum_{m_i,m_j} c_{\mathbf{n}} c_{\mathbf{n}_{ij}(m_i,m_j)} \left[- D^{\lambda_j}_{m_j n_j} \left.\frac{\partial D^{\lambda}_{m_i n_i}}{\partial \lambda}\right|_{\lambda=-\lambda_i} + \left.\frac{\partial D^{\lambda}_{m_i n_i}}{\partial \lambda}\right|_{\lambda=\lambda_i} D^{-\lambda_j}_{m_j n_j} \right] \\ &+ 2\omega A_i^2 \sum_{\mathbf{n}} \sqrt{n_i+1}\, c_{\mathbf{n}} c_{\mathbf{n}+\mathbf{e}_i} + 2(\omega\lambda_i + g) A_i^2 \sum_{\mathbf{n}} c_{\mathbf{n}}^2. \end{aligned} \tag{S57}$$

Here $j \in \mathrm{nn}(i)$ denotes nearest-neighbor sites of i.

The derivative with respect to the electronic coefficient A_i is

$$\begin{aligned}\frac{\partial \mathcal{E}_{\mathrm{H}}}{\partial A_i} = &- J \sum_{j\in\mathrm{nn}(i)} A_j \sum_{\mathbf{n}} \sum_{m_i,m_j} c_{\mathbf{n}} c_{\mathbf{n}_{ij}(m_i,m_j)} \left(D^{\lambda_i}_{m_i n_i} D^{-\lambda_j}_{m_j n_j} + D^{\lambda_j}_{m_j n_j} D^{-\lambda_i}_{m_i n_i} \right) \\ &+ 2\omega A_i \sum_{\mathbf{n}} \left(\sum_j n_j \right) c_{\mathbf{n}}^2 + 4(\omega\lambda_i + g) A_i \sum_{\mathbf{n}} \sqrt{n_i+1}\, c_{\mathbf{n}} c_{\mathbf{n}+\mathbf{e}_i} \\ &+ 2\lambda_i(\omega\lambda_i + 2g) A_i \sum_{\mathbf{n}} c_{\mathbf{n}}^2. \end{aligned} \tag{S58}$$

The derivative with respect to the phonon coefficient $c_{\mathbf{n}}$ is

$$\begin{aligned}\frac{\partial \mathcal{E}_{\mathrm{H}}}{\partial c_{\mathbf{n}}} =& - J \sum_{\langle i,j \rangle} A_i A_j \sum_{m_i,m_j} c_{\mathbf{n}_{ij}(m_i,m_j)} \left(D^{\lambda_i}_{m_i n_i} D^{-\lambda_j}_{m_j n_j} + D^{\lambda_j}_{m_j n_j} D^{-\lambda_i}_{m_i n_i} + D^{\lambda_i}_{n_i m_i} D^{-\lambda_j}_{n_j m_j} + D^{\lambda_j}_{n_j m_j} D^{-\lambda_i}_{n_i m_i} \right) \\ &+ 2\omega \left(\sum_i A_i^2 \right) \left(\sum_i n_i \right) c_{\mathbf{n}} + 2 \sum_i (\omega\lambda_i + g) A_i^2 \left[\sqrt{n_i + 1}\, c_{\mathbf{n}+\mathbf{e}_i} + \sqrt{n_i}\, c_{\mathbf{n}-\mathbf{e}_i} \right] \\ &+ 2 c_{\mathbf{n}} \sum_i \lambda_i (\omega\lambda_i + 2g) A_i^2. \end{aligned} \tag{S59}$$

The norm of the product state is

$$\mathcal{N}_{\mathrm{H}} = \langle \Phi_0 | \Phi_0 \rangle = \left(\sum_i A_i^2 \right) \left(\sum_{\mathbf{n}} c_{\mathbf{n}}^2 \right), \tag{S60}$$

and the normalized variational energy is

$$E^{\mathrm{H}}_{\mathrm{var}} = \frac{\mathcal{E}_{\mathrm{H}}}{\mathcal{N}_{\mathrm{H}}}. \tag{S61}$$

For any variational parameter $x \in \{\lambda_i, A_i, c_{\mathbf{n}}\}$,

$$\frac{\partial E^{\mathrm{H}}_{\mathrm{var}}}{\partial x} = \frac{\mathcal{N}_{\mathrm{H}}\, \partial_x \mathcal{E}_{\mathrm{H}} - \mathcal{E}_{\mathrm{H}}\, \partial_x \mathcal{N}_{\mathrm{H}}}{\mathcal{N}_{\mathrm{H}}^2}. \tag{S62}$$

The norm derivatives are

$$\frac{\partial \mathcal{N}_{\mathrm{H}}}{\partial A_i} = 2 A_i \sum_{\mathbf{n}} c_{\mathbf{n}}^2, \qquad \frac{\partial \mathcal{N}_{\mathrm{H}}}{\partial c_{\mathbf{n}}} = 2 c_{\mathbf{n}} \sum_i A_i^2, \qquad \frac{\partial \mathcal{N}_{\mathrm{H}}}{\partial \lambda_i} = 0. \tag{S63}$$

After each gradient-descent step, the coefficients are normalized so that $\sum_i A_i^2 = 1$ and $\sum_{\mathbf{n}} c_{\mathbf{n}}^2 = 1$.

S3.3 Exact diagonalization in the displaced frame

For exact diagonalization, it is convenient to set $\lambda_i = -g/\omega$, which cancels the linear electron–phonon coupling in the transformed frame. Equation (S51) then becomes

$$\begin{aligned}\hat{H}_{\mathrm{H}}(\lambda_i = -g/\omega) =& - J \sum_{\langle i,j \rangle} \left[\hat{c}_i^\dagger \hat{c}_j e^{(g/\omega)(\hat{b}_i^\dagger - \hat{b}_i)} e^{-(g/\omega)(\hat{b}_j^\dagger - \hat{b}_j)} + \hat{c}_j^\dagger \hat{c}_i e^{(g/\omega)(\hat{b}_j^\dagger - \hat{b}_j)} e^{-(g/\omega)(\hat{b}_i^\dagger - \hat{b}_i)} \right] \\ &+ \omega \sum_i \hat{b}_i^\dagger \hat{b}_i - \frac{g^2}{\omega} \sum_i \hat{c}_i^\dagger \hat{c}_i \hat{c}_i^\dagger \hat{c}_i \\ &\equiv \hat{H}^{(1)}_{\mathrm{H}} + \hat{H}^{(2)}_{\mathrm{H}} + \hat{H}^{(3)}_{\mathrm{H}}. \end{aligned} \tag{S64}$$

The diagonalization basis is $|\mathbf{n}; i\rangle = |n_1, \ldots, n_{N_{\mathrm{site}}}\rangle \otimes |i\rangle$ with $0 \le n_j \le N_{\mathrm{cut}}$ and $1 \le i \le N_{\mathrm{site}}$. It may be encoded by the integer label

$$r = 1 + (i-1)(N_{\mathrm{cut}} + 1)^{N_{\mathrm{site}}} + \sum_{j=1}^{N_{\mathrm{site}}} n_j (N_{\mathrm{cut}} + 1)^{j-1}. \tag{S65}$$

The nonzero matrix elements are

$$\langle \mathbf{n}; i| \hat{H}_{\mathrm{H}}^{(1)} |\mathbf{m}; j\rangle = -J D^{g/\omega}_{n_i m_i} D^{-g/\omega}_{n_j m_j}, \tag{S66}$$

only for nearest-neighbor sites (i, j) and $n_k = m_k$ for $k \neq i, j$;

$$\langle \mathbf{n}; i| \hat{H}_{\mathrm{H}}^{(2)} |\mathbf{m}; j\rangle = \omega \sum_k m_k, \tag{S67}$$

only for $i = j$ and $n_k = m_k$ for all k; and

$$\langle \mathbf{n}; i| \hat{H}_{\mathrm{H}}^{(3)} |\mathbf{m}; j\rangle = -\frac{g^2}{\omega}, \tag{S68}$$

again only for $i = j$ and $n_k = m_k$ for all k. The identity $D^{\lambda}_{nm} = D^{-\lambda}_{mn}$ makes the Hamiltonian matrix real and symmetric.

S3.4 Second-order perturbation theory for the Holstein transformed Hamiltonian

We start from the transformed Holstein Hamiltonian in Eq. (S51). The optimized zeroth-order electronic and phononic states are

$$\left|\Phi_0^{\mathrm{E}}\right\rangle = \sum_{i=1}^{N_{\mathrm{site}}} A_i \left|i\right\rangle, \qquad \left|\Phi_0^{\mathrm{P}}\right\rangle = \sum_{\mathbf{n}} c_{\mathbf{n}} \left|\mathbf{n}\right\rangle, \tag{S69}$$

and

$$\left|\Phi_0\right\rangle = \left|\Phi_0^{\mathrm{E}}\right\rangle \otimes \left|\Phi_0^{\mathrm{P}}\right\rangle. \tag{S70}$$

To construct the zeroth-order Hamiltonian for perturbation theory, we define the electronic and phononic mean-field Hamiltonians by partial expectation values of the transformed Hamiltonian:

$$\hat{H}_{\mathrm{E}} = \left\langle \Phi_0^{\mathrm{P}}\right| \hat{H}_{\mathrm{H}}(\boldsymbol{\lambda}) \left|\Phi_0^{\mathrm{P}}\right\rangle, \tag{S71}$$

$$\hat{H}_{\mathrm{P}} = \left\langle \Phi_0^{\mathrm{E}}\right| \hat{H}_{\mathrm{H}}(\boldsymbol{\lambda}) \left|\Phi_0^{\mathrm{E}}\right\rangle. \tag{S72}$$

We define the displaced-phonon overlap

$$\mathcal{K}_{ij} = \left\langle \Phi_0^{\mathrm{P}}\right| e^{-\lambda_i(\hat{b}_i^\dagger - \hat{b}_i)} e^{\lambda_j(\hat{b}_j^\dagger - \hat{b}_j)} \left|\Phi_0^{\mathrm{P}}\right\rangle, \tag{S73}$$

or explicitly,

$$\mathcal{K}_{ij} = \sum_{\mathbf{n}} \sum_{m_i, m_j} c_{\mathbf{n}} c_{\mathbf{n}_{ij}(m_i, m_j)} D^{-\lambda_i}_{n_i m_i} D^{\lambda_j}_{n_j m_j}. \tag{S74}$$

With these definitions, the electronic mean-field Hamiltonian becomes

$$\begin{aligned}\hat{H}_{\mathrm{E}} = &- J \sum_{\langle i,j\rangle} \left(\mathcal{K}_{ij} \hat{c}_i^\dagger \hat{c}_j + \mathcal{K}_{ji} \hat{c}_j^\dagger \hat{c}_i\right) + 2 \sum_i (\omega\lambda_i + g) \left(\sum_{\mathbf{n}} \sqrt{n_i + 1}\, c_{\mathbf{n}} c_{\mathbf{n}+\mathbf{e}_i}\right) \hat{c}_i^\dagger \hat{c}_i \\ &+ \sum_i \lambda_i(\omega\lambda_i + 2g) \hat{c}_i^\dagger \hat{c}_i \hat{c}_i^\dagger \hat{c}_i + \omega \sum_{\mathbf{n}} \left(\sum_i n_i\right) c_{\mathbf{n}}^2.\end{aligned} \tag{S75}$$

For a single electron, $\hat{c}_i^\dagger \hat{c}_i \hat{c}_i^\dagger \hat{c}_i = \hat{c}_i^\dagger \hat{c}_i$ within the one-particle sector. Similarly, the phononic mean-field Hamiltonian is

$$\begin{aligned}\hat{H}_\mathrm{P} = &- J \sum_{\langle i,j \rangle} A_i A_j \left[e^{-\lambda_i (\hat{b}_i^\dagger - \hat{b}_i)} e^{\lambda_j (\hat{b}_j^\dagger - \hat{b}_j)} + e^{-\lambda_j (\hat{b}_j^\dagger - \hat{b}_j)} e^{\lambda_i (\hat{b}_i^\dagger - \hat{b}_i)} \right] + \omega \sum_i \hat{b}_i^\dagger \hat{b}_i \\ &+ \sum_i (\omega \lambda_i + g) A_i^2 \left(\hat{b}_i^\dagger + \hat{b}_i \right) + \sum_i \lambda_i (\omega \lambda_i + 2g) A_i^2. \end{aligned} \tag{S76}$$

The zeroth-order Hamiltonian is

$$\hat{H}_0^\mathrm{H} = \hat{H}_\mathrm{E} + \hat{H}_\mathrm{P} - E_0, \qquad E_0 = \langle \Phi_0 | \, \hat{H}_\mathrm{H}(\boldsymbol{\lambda}) \, | \Phi_0 \rangle \,, \tag{S77}$$

for which

$$\hat{H}_0^\mathrm{H} \, | \Phi_0 \rangle = E_0 \, | \Phi_0 \rangle \,. \tag{S78}$$

This follows from the variational stationarity of E_0 with respect to A_i and $c_\mathbf{n}$.

Let

$$\hat{H}_\mathrm{E} \left| \Phi_p^\mathrm{E} \right\rangle = \epsilon_p^\mathrm{E} \left| \Phi_p^\mathrm{E} \right\rangle, \qquad \hat{H}_\mathrm{P} \left| \Phi_q^\mathrm{P} \right\rangle = \epsilon_q^\mathrm{P} \left| \Phi_q^\mathrm{P} \right\rangle. \tag{S79}$$

Then

$$| \Phi_{pq} \rangle = \left| \Phi_p^\mathrm{E} \right\rangle \otimes \left| \Phi_q^\mathrm{P} \right\rangle, \qquad E_{pq} = \epsilon_p^\mathrm{E} + \epsilon_q^\mathrm{P} - E_0. \tag{S80}$$

The residual Hamiltonian is

$$\delta \hat{H}^\mathrm{H} = \hat{H}_\mathrm{H}(\boldsymbol{\lambda}) - \hat{H}_0^\mathrm{H}. \tag{S81}$$

The second-order energy correction and first-order corrected state are

$$E_0^{(2)} = \sum_{(p,q) \neq (0,0)} \frac{| \langle \Phi_{pq} | \, \delta \hat{H}^\mathrm{H} \, | \Phi_{00} \rangle |^2}{E_{00} - E_{pq}}, \tag{S82}$$

$$\left| \psi_0^{(1)} \right\rangle = | \Phi_{00} \rangle + \sum_{(p,q) \neq (0,0)} \frac{\langle \Phi_{pq} | \, \delta \hat{H}^\mathrm{H} \, | \Phi_{00} \rangle}{E_{00} - E_{pq}} \, | \Phi_{pq} \rangle \,. \tag{S83}$$

If $|\psi_\mathrm{ex}\rangle$ is the exact ground state of the transformed Hamiltonian, the fidelity diagnostic used in the main text is

$$F_\mathrm{var} = | \langle \psi_\mathrm{ex} | \Phi_0 \rangle |^2, \qquad F_\mathrm{2nd} = \frac{\left| \left\langle \psi_\mathrm{ex} \middle| \psi_0^{(1)} \right\rangle \right|^2}{\left\langle \psi_0^{(1)} \middle| \psi_0^{(1)} \right\rangle}. \tag{S84}$$

These quantities measure how close the optimized product state and the perturbatively corrected state are to the exact ground state in the transformed frame.

S4 Additional Computational Details, Convergence Protocols, and Diagnostics

S4.1 Parameter counts and scaling

For a matter basis of dimension $N_{\rm M}$ and a retained bosonic Hilbert space of dimension $D_{\rm B}$, the product-state ansatz in the transformed frame contains $N_{\rm M}$ matter coefficients, $D_{\rm B}$ bosonic coefficients, and the displacement parameters $\lambda_{\alpha,\mu}$, subject to normalization and gauge choices. The displaced-oscillator overlaps D^{λ}_{nm} are evaluated analytically using Eqs. (S30) and (S31). In numerical implementations, these overlaps can be reused for a given set of displacements and cutoff, so the dominant cost is the contraction of transformed-Hamiltonian matrix elements in the retained product basis.

Table 1: Parameter counts for the benchmark models. Here $D_{\rm B}$ denotes the retained bosonic Hilbert-space dimension.

Model	Matter dimension	Bosonic dimension and displacements
Dicke	$N_{\rm M} = N + 1$	$D_{\rm B} = N_{\rm cut} + 1$, $N + 1$ displacements λ_l
Two-site Holstein	$N_{\rm M} = 2$	$D_{\rm B} = (N_{\rm cut} + 1)^2$, two displacements λ_i
Symmetric Holstein	$N_{\rm M} = 2$	same $D_{\rm B}$, with symmetry constraints on λ_i and coefficients

S4.2 Bosonic cutoff convergence

The cutoff $N_{\rm cut}$ should be increased until the zeroth-order variational energy, the second-order correction, and the relevant observables become insensitive to further enlargement of the retained Fock space. A practical convergence check is

$$\Delta E_{\rm var}(N_{\rm cut}) = \left|E_{\rm var}(N_{\rm cut}) - E_{\rm var}(N_{\rm cut} + \Delta N)\right|, \tag{S85}$$

$$\Delta E^{(2)}(N_{\rm cut}) = \left|E^{(2)}(N_{\rm cut}) - E^{(2)}(N_{\rm cut} + \Delta N)\right|. \tag{S86}$$

Convergence of the optimized displacement parameters and observables such as photon or phonon occupation provides an additional check. This procedure is especially important in the intermediate-coupling regime, where residual non-vacuum bosonic excitations are largest.

Table 2 summarizes the cutoff convergence for the two representative benchmarks. The Dicke calculation for $N = 1$ converges systematically as the photon cutoff is increased from $N_{\rm cut} = 4$ to 20. The maximum second-order energy error decreases from 1.328×10^{-2} to 2.33×10^{-3}, with only a negligible change between $N_{\rm cut} = 16$ and 20. The two-site Holstein benchmark shows similar convergence: the maximum second-order energy error decreases from 1.261×10^{-2} at $N_{\rm cut} = 4$ to 4.88×10^{-3} at $N_{\rm cut} = 16$, with the results already nearly saturated at $N_{\rm cut} = 12$. These data support the cutoff choices used in the main-text benchmarks and show that the remaining errors are not dominated by bosonic Hilbert-space truncation.

S4.3 Transformed-vacuum limit

The transformed-vacuum approximation is obtained as a restricted case of the present ansatz by fixing the bosonic factor to the oscillator vacuum in the transformed frame. For the Dicke model this corresponds to

$$|\Phi_0^B\rangle = |0\rangle, \qquad c_0 = 1, \qquad c_{n>0} = 0, \tag{S87}$$

Table 2: Bosonic cutoff convergence of the maximum relative ground-state energy error for the optimized non-vacuum bosonic ansatz. The labels "var" and "2nd" denote the zeroth-order variational result and the result including the second-order perturbative correction, respectively.

Model	N_{cut}	Max. error (var)	Max. error (2nd)
Dicke, $N = 1$	4	1.715×10^{-2}	1.328×10^{-2}
Dicke, $N = 1$	8	1.275×10^{-2}	5.56×10^{-3}
Dicke, $N = 1$	12	1.254×10^{-2}	3.16×10^{-3}
Dicke, $N = 1$	16	1.252×10^{-2}	2.34×10^{-3}
Dicke, $N = 1$	20	1.251×10^{-2}	2.33×10^{-3}
Holstein	4	1.913×10^{-2}	1.261×10^{-2}
Holstein	8	1.445×10^{-2}	5.04×10^{-3}
Holstein	12	1.425×10^{-2}	4.89×10^{-3}
Holstein	16	1.423×10^{-2}	4.88×10^{-3}

whereas for the multimode Holstein model it corresponds to

$$|\Phi_0^P\rangle = |0, 0, \ldots, 0\rangle. \tag{S88}$$

Comparing this restricted ansatz with the optimized non-vacuum bosonic wave function provides a direct diagnostic of the importance of residual bosonic excitations that remain after the state-dependent displacement. The transformed-vacuum approximation is expected to become accurate in the asymptotic strong-coupling limit, but it can be less accurate in the intermediate regime where the displacement does not fully remove residual matter–boson correlations. The cutoff-convergence results in Table 2 demonstrate that the improvement obtained by the optimized non-vacuum ansatz is not a cutoff artifact: once the retained Fock space is sufficiently enlarged, the maximum errors are saturated while remaining much smaller than those of the transformed-vacuum restriction reported in Table 3.

Table 3 summarizes this comparison for the Dicke and Holstein benchmarks. For the Dicke model, optimizing the non-vacuum bosonic state reduces the maximum second-order energy error from 3.467×10^{-2} to 2.33×10^{-3} for $N = 1$, from 4.46×10^{-3} to 3.303×10^{-4} for $N = 10$, and from 6.3229×10^{-4} to 2.49257×10^{-5} for $N = 100$. For the Holstein benchmark, the corresponding maximum second-order error decreases from 8.996×10^{-2} to 4.88×10^{-3}. These reductions show that the optimized residual bosonic wave function is particularly important in the intermediate-coupling regime, where the state-dependent displacement alone does not fully remove non-vacuum bosonic components.

S4.4 Low-frequency or sluggish-boson regime

Low-frequency bosonic modes are more demanding because the displacement scale g/ω increases and more bosonic quanta may contribute in a bare representation. In such cases, the retained Fock space should be enlarged systematically and the perturbative correction should be monitored. A useful dimensionless indicator is

$$\eta_E = \frac{|E^{(2)}|}{|E_{\mathrm{var}}|}. \tag{S89}$$

A growing value of η_E signals that residual transformed-frame coupling is becoming less perturbative and that either a larger bosonic basis or a more flexible zeroth-order ansatz may be required. This criterion complements direct cutoff convergence of E_{var}, $E^{(2)}$, and relevant observables.

Table 3: Maximum relative ground-state energy error obtained with the transformed-vacuum restriction and with the optimized non-vacuum bosonic state. "var" denotes the zeroth-order variational result, whereas "2nd" includes the second-order perturbative correction.

Benchmark	Bosonic ansatz	var	2nd
Dicke, $N = 1$	transformed vacuum	4.197×10^{-2}	3.467×10^{-2}
Dicke, $N = 1$	optimized non-vacuum	1.251×10^{-2}	2.33×10^{-3}
Dicke, $N = 10$	transformed vacuum	5.88×10^{-3}	4.46×10^{-3}
Dicke, $N = 10$	optimized non-vacuum	2.11×10^{-3}	3.303×10^{-4}
Dicke, $N = 100$	transformed vacuum	6.9708×10^{-4}	6.3229×10^{-4}
Dicke, $N = 100$	optimized non-vacuum	1.78059×10^{-4}	2.49257×10^{-5}
Holstein	transformed vacuum	1.0099×10^{-1}	8.996×10^{-2}
Holstein	optimized non-vacuum	1.422×10^{-2}	4.88×10^{-3}

Table 4 summarizes an explicit low-frequency test for the two-site Holstein benchmark. The baseline calculation uses $\omega/J = 1$, while the sluggish-boson case uses $\omega/J = 0.5$. When the boson frequency is reduced, the maximum variational energy error increases from 1.423×10^{-2} to 2.598×10^{-2}, and the maximum second-order error increases from 4.88×10^{-3} to 1.277×10^{-2}. The minimum fidelity decreases from 0.98903 to 0.96237 at the variational level and from 0.99586 to 0.97319 after the second-order correction. These results are consistent with the expectation that sluggish bosons provide a more demanding regime for transformed-frame perturbation theory. At the same time, the second-order correction still improves both the maximum energy error and the minimum fidelity at $\omega/J = 0.5$, indicating that the optimized dressed reference remains useful, although the residual coupling is less perturbative than for $\omega/J = 1$.

Table 4: Low-frequency Holstein benchmark. The table compares the maximum relative ground-state energy error and the minimum fidelity for the optimized non-vacuum bosonic ansatz at $\omega/J = 1$ and $\omega/J = 0.5$.

ω/J	Max. error (var)	Max. error (2nd)	Min. fidelity (var)	Min. fidelity (2nd)
1	1.423×10^{-2}	4.88×10^{-3}	0.98903	0.99586
0.5	2.598×10^{-2}	1.277×10^{-2}	0.96237	0.97319

S4.5 Diagnostics when exact diagonalization is unavailable

When an exact solution is available, the fidelity used in the main text is the squared overlap between the exact transformed-frame ground state and the approximate state. For the perturbatively corrected state, Eq. (S84) includes the first-order wave-function correction. When exact diagonalization is unavailable, the following diagnostics can be used instead: convergence with respect to N_{cut}; the relative correction η_E; the norm of the first-order correction,

$$\eta_\psi = \left[\sum_{\nu \neq 0} \left| \frac{\langle \Phi_\nu | \delta \hat{H} | \Phi_0 \rangle}{E_0 - E_\nu} \right|^2 \right]^{1/2} ; \tag{S90}$$

stability of the optimized branch with respect to initial conditions; smooth variation of the solution under small parameter changes; and consistency of observables. These diagnostics are particularly useful near intermediate coupling, where the residual matter–boson correlations are largest and the variational energy alone may not identify the best physical state.
